# A feasibility study to assess non-clinical community health workers’ capacity to use simplified protocols and tools to treat severe acute malnutrition in Niger state Nigeria

**DOI:** 10.1186/s12913-021-07118-4

**Published:** 2021-10-15

**Authors:** Olatunde Adesoro, Olusola Oresanya, Helen Counihan, Prudence Hamade, Dare Eguavon, Chika Emebo, Bethany Marron, Naoko Kozuki, Amina Isah, Patrick Gimba, Chris Osa Isokpunwu, Kolawole Maxwell, James K. Tibenderana

**Affiliations:** 1 Malaria Consortium, 33 Pope John Paul Street, Maitama, Abuja, Nigeria; 2 Malaria Consortium, London, UK; 3 International Rescue Committee, New York, USA; 4 Niger State Ministry of Health, Minna, Nigeria; 5 Federal Ministry of Health, Abuja, Nigeria

**Keywords:** Severe acute malnutrition, Non-clinical community health workers, Simplified protocols, Integrated community case management

## Abstract

**Background:**

Severe acute malnutrition (SAM) is a major determinant of childhood mortality and morbidity. Although integrated community case management (iCCM) of childhood illnesses is a strategy for increasing access to life-saving treatment, malnutrition is not properly addressed in the guidelines. This study aimed to determine whether non-clinical Community Health Workers (called Community-Oriented Resource Persons, CORPs) implementing iCCM could use simplified tools to treat uncomplicated SAM.

**Methods:**

The study used a sequential multi-method design and was conducted between July 2017 and May 2018. Sixty CORPs already providing iCCM services were trained and deployed in their communities with the target of enrolling 290 SAM cases. Competency of CORPs to treat and the treatment outcomes of enrolled children were documented. SAM cases with MUAC of 9 cm to < 11.5 cm without medical complications were treated for up to 12 weeks. Full recovery was at MUAC≥12.5 cm for two consecutive weeks. Supervision and quantitative data capturing were done weekly while qualitative data were collected after the intervention.

**Results:**

CORPs scored 93.1% on first assessment and increment of 0.11 (95% CI, 0.05–0.18) points per additional supervision conducted. The cure rate from SAM to full recovery, excluding referrals from the denominator in line with the standard for reporting SAM recovery rates, was 73.5% and the median length of treatment was 7 weeks. SAM cases enrolled at 9 cm to < 10.25 cm MUAC had 31% less likelihood of recovery compared to those enrolled at 10.25 cm to < 11.5 cm. CORPs were not burdened by the integration of SAM into iCCM and felt motivated by children’s recovery. Operational challenges like bad terrains for supervision, supply chain management and referrals were reported by supervisors, while Government funding was identified as key for sustainability.

**Conclusion:**

The study demonstrated that with training and supportive supervision, CORPs in Nigeria can treat SAM among under-fives, and refer complicated cases using simplified protocols as part of an iCCM programme. This approach seemed acceptable to all stakeholders, however, the effect of the extra workload of integrating SAM into iCCM on the quality of care provided by the CORPs should be assessed further.

**Supplementary Information:**

The online version contains supplementary material available at 10.1186/s12913-021-07118-4.

## Introduction

Severe acute malnutrition (SAM) is a major determinant of mortality and morbidity among children under the age of 5 years ^(1)^. It increases the risk of death from common childhood illnesses such as pneumonia, diarrhoea and malaria, contributing to about 45% of deaths in this age group [1]. In Nigeria, according to the National Demographic and Health Survey (NDHS) carried out in 2018, 37% of children age 6–59 months are stunted (short for their age), 7% are wasted (underweight for their height: Moderate Acute Malnutrition, MAM = 5% and Severe Acute Malnutrition, SAM = 2%), 22% are underweight (thin for their age), and 2% are overweight (heavy for their height). Parental nutrition care is also poor as only 29% of children under age 6 months are exclusively breastfed for 6 months post birth and only 11% of children age 6–23 months were fed a minimum acceptable diet [2].

Integrated Community Case Management (iCCM) is a community-based intervention that seeks to achieve equitable access to healthcare services by complimenting and extending public health services to medically underserved communities. It focusses on children under the age of 5 years, providing timely and effective diagnosis and treatment of the three most common childhood illnesses of malaria, diarrhoea and pneumonia [3]. Since 2015, Malaria Consortium has been operating iCCM projects in Niger State that train community health workers (CHWs) – also known as community-oriented resource persons (CORPs) in Nigeria – to treat children under 5 years for malaria, diarrhoea and pneumonia in their homes, and to diagnose and refer cases of severe illness to health facilities. The CORPs operate as part of the primary health care system whereby each CORP is attached to a primary healthcare facility and assigned a healthcare worker in the health facility as supervisor. The supervisors provided mentorship and health commodity management services in support of the CORPs.

Although iCCM is recognised as a strategy for increasing access to life-saving treatment for childhood illnesses, malnutrition is not currently adequately addressed in the Nigeria national iCCM guidelines. Rather CORPs are meant to just screen for SAM and then refer to health facilities for treatment. The Nigeria’s National Policy on Food and Nutrition advocates that management of acute malnutrition, including stabilisation centres addressing the needs of severely ill children with SAM, should be located at health facility level, where personnel with requisite skills and qualifications are be available [4, 5]. Completing referral for patients with SAM remains a challenge, particularly as the very few facilities that offer care for SAM are located far from many rural communities, where most of the affected children live. In Niger state and most of the states in Nigeria, this model of delivery of SAM treatment is hampered by weak infrastructure, poorly trained staff and inadequate supplies, which are also barriers for households as observed in similar programmes in other countries [6]. This is coupled with the challenge of poor access to care due to remote communities’ distance from healthcare providers and other high opportunity costs to seeking treatment [7].

Providing patients with SAM treatment using the ready-to-use therapeutic food (RUTF) in their own communities, alongside care for other childhood diseases, could improve treatment coverage and reduce the number of children defaulting from treatment which typically takes 8–12 weeks of weekly visits to the health facility before the treatment course is completed [8]. Promising and cost-effective models for community-level treatment of SAM exist and has been demonstrated in other African countries [9], however, adapting these for non-clinical personnel settings has not been studied in Nigeria. This will provide evidence on the safety, effectiveness and acceptability of the models for community-level treatment of SAM cases.

Treatment of SAM cases by CORPs providing iCCM services, is however not without concerns. There are anecdotal fears about the safety of the children due to the limited training and literacy levels of the CORPs, extra workload for the CORPs and effects of adding SAM treatment to the iCCM protocols on the quality of care provided. However, there is evidence that with minimal training, CORPs are able to appropriately treat SAM in the community without compromising treatment outcomes and this can lead to improved access to treatment [10, 11].

Therefore, in 2017, Malaria Consortium and International Rescue Committee (IRC) contributed to a consortium of implementing partners to test a simplified protocol and tools in four contexts with a goal to determine whether non-clinical CORPs can treat uncomplicated SAM cases using a simplified protocol and tools integrated into iCCM treatment algorithm without medical complications. IRC had previously developed, tested and adapted these innovative and simplified tools in South Sudan [12]. The human centred design approach was used for the adaptation of the protocol and tools in different context across Africa (Kenya, Malawi, Mali, Nigeria and South Sudan) and piloted to develop an evidence base through a global coalition.

Here presented are the findings from the study conducted in Niger State, Nigeria by Malaria Consortium between July 2017 and May 2018, which focused on the competency of CORPs in following the protocol, effectiveness of the intervention in curing children with SAM and the acceptability of the approach amongst key stakeholders.

## Methods

### Study design

Using a sequential multi-method design [13], the capacity of non-clinical CORPs to use a simplified protocol for the treatment of SAM at the community level and the treatment outcomes of children who received treatment through this program were assessed and the findings complemented with opinions of key stakeholders on the acceptability of the treatment approach.

### Target population

The study was implemented in Mariga and Rijau, two of the six local government areas (LGAs) in Niger state where iCCM was being implemented at the time with a projected total under-five population of 104,912 [14]. Most families in these communities are poor, illiterate and lack access to basic social amenities and health care. The selection of the LGAs was informed by an unpublished report of a mass screening activity for malnutrition carried out in the state in 2016 which found prevalence of SAM to be 10% in the project sites [15].

### Contextualisation of protocol and tools

The simplified SAM treatment protocol and tools used in South Sudan in 2016 [16] were pretested by officials of Niger State Ministry of Health, Malaria Consortium and IRC project staff with selected CORPs in Niger State in July 2017 and adapted based on their feedback, particularly the mid-upper arm circumference (MUAC) tape, weighing scale, calculator and patient register. Thus, a series of revisions were effected to make the tools more user-friendly, compliant with the cultural context of the study areas and Nigerian SAM treatment protocol. The MUAC tape is a colour-coded malnutrition screening tool that measures the circumference of the left upper arm at the mid-point between the tips of the shoulder and elbow of a child. A child with MUAC on the green zone is well-nourished while yellow zone indicates moderate acute malnutrition (MAM) and the red zone indicates severe acute malnutrition (SAM). The red (SAM) zone on the simplified MUAC used for the South Sudan study was further divided into three (pink, bright red and deep red) in order of severity. The weighing scale determines the weight and corresponding daily quantity of RUTF needed for treatment. The weighing scale is calibrated into red (referral) and other zones with dots corresponding to number of RUTF sachets needed for daily treatment. The calculator helps to determine the weekly number of RUTF and the register is used for documenting bio-data and treatment date for the patient. Key revisions on the tools included an amendment of the colours of the MUAC tape and weighing scale by changing referral colours to ‘bright red’ to align with iCCM colour codes for danger signs and referrals; alignment of the treatment duration with the national community-based management of acute malnutrition (CMAM) guidelines that has maximum treatment period of 12 weeks instead of 16 weeks contained in the South Sudan protocol; and use of context-specific images in the simplified protocol. (Kindly refer to the supplementary files for more details on algorithm, tools and graphics.)

### Sample size and CHW selection

A minimum of 290 children were expected to be treated by 60 CORPs over a period of 6 months based on an estimation using the Sphere Humanitarian Standard (SHS) of 75% recovery rate for SAM. A one-sample non-inferiority test was carried against this rate with the assumption that a rate that is not more than an absolute 10% lower than the SHS would be considered non-inferior, with an alpha value of 0.05, a power of 0.90 and a loss to-follow-up rate of 10%. The latter was defined as loss to follow-up caused by the supply side (e.g. CORPs dropping out of the programme or stock-outs of ready-to-use therapeutic food (RUTF)).

CORPs already trained on iCCM and providing treatment services in their homes for malaria, diarrhoea and pneumonia as volunteers in hard-to-reach communities for a minimum of 2 years, were selected and trained on the simplified protocol. Each CORP had between 115 and 200 under-five children within the catchment area. The CORPs were trained in batches over 6 days with a trainer to trainee ratio of 1:3 and strong focus on the use of the simplified SAM protocol and its integration with the iCCM treatment algorithm. CORPs’ subsequent participation in the pilot was determined by a post-training assessment score of 80 % and more. Weekly supervision was provided by community health extension workers (CHEWs) who were based at the primary healthcare facilities and already iCCM supervisors. The supervisors were also trained and in turn supervised by the LGAs’ health teams.

### Supply chain management

A total of 400 cartons of RUTF, additional doses of amoxicillin and albendazole were procured and distributed to CORPs for treating SAM cases. Commodity logistics was built on the existing iCCM commodity supply mechanism, however, to store the RUTF securely, metal boxes were provided to CORPs. Supervisors were responsible for monitoring stock levels and restocking the CORPs.

### Enrolment, treatment and discharge of SAM cases

Prior to commencement of the intervention, the demand creation activities of iCCM were strengthened to sensitise members of communities on the additional nutrition services available with the CORPs. Informed consent was received from caregivers accessing iCCM services and whose children were malnourished. Admission to CORP’s nutrition treatment was based on a modified colour-coded MUAC strip. Children were assessed using the iCCM algorithm to either identify danger signs and refer, or treat based on the protocol. Any child screened and found not to have any iCCM danger signs but who fell in the severe ‘malnutrition zone’ of the MUAC (red or pink), was given an appetite test to further screen for enrolment eligibility. If the child passed the appetite test by being able to eat as much as one quarter of the RUTF pack, s/he was enrolled in the study. A failed appetite test resulted in referral to the appropriate health facilities for treatment.

Upon enrolment, CORPs administered amoxicillin and albendazole to each SAM case according to the simplified protocol. To determine the RUTF doses required per day, the child was weighed with the Salter scale overlaid with a dosage chart that guided the CORP on the number of sachets of RUFT to administer, depending on where the weight indicator fell on the chart. Rather than reading weight as figures in kilograms, the scale indicated the number of sachets of RUTF needed per day. The seven-day dosage was then calculated with the aid of the simplified calculator, a rectangular strip with 7 pockets, one for each day dose.

Using a flipchart, CORPs counselled caregivers on how to administer the RUTF and other medications at home, adhere to the daily dosage, maintain good hygiene and return the following week to continue treatment, except if the condition of child got worse before the next appointment. Each SAM patient encounter was recorded in a register and CORPs followed up defaulting enrolees with home visits, recorded children’s progress every week and discharged as appropriate based on possible outcomes listed in Table 1. The maximum treatment period for any admitted case was 12 weeks.

**Table 1:** Admission and discharge criteria and associated treatment actions

**Admission criteria**	
**MUAC colour codes**	**CORP’s Action**
Red: < 9 cm	Refer to nearest nutrition clinic – likely to need inpatient care
Dark red: 9 < 10.5 cm	Treatment by CORP if the child passes the appetite test
Pink: 10.25 - < 11.5 cm	Treatment by CORP if the child passes the appetite test
Yellow: 11.5 to < 12.5 cm	Nutrition counselling
Green: > 12.5 cm	No nutrition treatment
**Discharge criteria**	
**Criteria**	**CORP’s Action**
MUAC colour Red	**Refer** to health facility
MUAC colour two greens in a row	Recovered, **DISCHARGE**
Two missed visits in a row	Defaulted, **DISCHARGE**
MUAC is below admission MUAC reading	Deteriorated, **refer, DISCHARGE**
If by 12th week never had two greens in a row	Non-response, **refer, DISCHARGE**
Otherwise	Continue treatment

### Data collection and analysis

CORPs’ demographic information was collected at the outset of the project by MC Research Officers, while children’s demographic information was collected by the CORPs on patient register and by supervisors on supervision checklist used for assessing compliance of CHWs with the simplified treatment protocol. Thereafter, progress data for enrolees (i.e. malnutrition status, number of weeks in treatment and treatment outcomes) and stock levels of commodities were collected using treatment registers and stock management tools respectively. Each CORP was supervised and assessed weekly by their supervisors on ability to use the simplified tools and compliance with the treatment protocol. There was an average of three CORPs to one supervisor.

Data analysis focused on treatment outcomes (% recovered, % defaulted, % non-response, % death), and number of weeks in treatment and data was stratified by child background characteristics including age and severity of malnutrition at enrolment. Single and multiple regression models were run to determine associations between child/CORP characteristics and treatment outcomes (recovered vs. not recovered as reference group) using a modified Poisson approach to arrive at an appropriate risk ratio estimate, which is more interpretable than odds ratio [17]. Stata version 14 was used for analysis.

The qualitative component of the study sought to probe for in-depth information from the stakeholders on the acceptability of the intervention. A total of eight Focus Group Discussions (FGDs) were conducted. There were four FGDs conducted with CORPs, two with supervisors and two with caregivers. Study communities were clustered into four groups based on contiguity and participants for each FGD were randomly selected from each cluster. Similarly, a total number of 17 in-depth interviews (IDIs) were conducted with CORPs; caregivers; policy makers and programme implementers. The respondents for the IDI were CORPs highest and lowest performers based on assessment scores while the IDI respondents among caregivers were those with cured, defaulted, non-response and referred treatment outcomes. The policy makers for IDI were from the National, State and LGA levels while programme managers were from both Government and non-Government agencies.

### Quantitative results

#### Demographic characteristics of CORPs

As Table 2 shows, roughly equal numbers of CORPs were assessed in the two LGAs, the vast majority of whom were male and aged 18–35 years. A large proportion reported they had senior secondary level education (not verified), could read without any difficulty and had been working as CORPs for between three and 4 years.

**Table 2:** Background and demographic characteristics of community-oriented resource persons

***n*** = 67	
LGA	
Mariga	34 (50.7)
Rijau	33 (49.3)
**Sex**	
Male	64 (95.5%)
Female	3 (4.5%)
**Age (years)**	
18–35	45 (67.1%)
36–50	15 (22.4%)
50–60	6 (9%)
60+	1 (1.5%)
**Education**	
Primary	5 (7.5%)
Junior Secondary	8 (11.9%)
Senior Secondary	47 (70.2%)
Others e.g. Koranic education	7 (10.4%)
**Ability to read**	
With some difficulty	21 (31.3%)
Without difficulty	46 (68.7%)
**Experience working as a CORP (years)**	
1–2	26 (38.8%)
3–4	41 (61.2%)

#### Performance of CHWs

There were 528 supervisory visits and performance assessments on the 60 CORPs who treated SAM cases. All (100%) CORPs with current SAM cases were supervised weekly as planned. The mean number of assessment carried out on each CORP was 10; median 10, range 1–28, IQR was 5–15. CORP performance was analysed from a maximum of twelve consecutive competency-based assessment scores. Tables 3, 4 and 5 show the performance of the CORPs. First competency-based assessment was high at 93.1% with an increase of 0.11 (95% CI: 0.05. 0.18) points in score per additional supervision conducted. When CORPs’ performance on individual tasks in the treatment procedure was examined (Table 4), all CORPs scored more than 90 % in all tasks except prescription and recording of albendazole where the score was 82.2% (95% Ci: 71.4, 89.5). All the CORPs did excellently well in terms of giving adequate messages to caregiver on RUTF dosage Table 5).

**Table 3:** Performance score of CORP on biodata and screening for danger signs, accounting for clustering at CORP level

Procedure	Score (95% CI)
Recording sex and age on register	99.3 (95.2, 99.9)
Identification of correct register page	99.2 (97.9, 99.7)
Screening for cough	98.1 (96.4, 99.0)
Screening for diarrhoea	99.0 (97.0, 99.7)
Screening for fever	98.6 (97.3, 99.2)
Screening for blood or worms in stool	95.2 (91.9, 97.2)
Screening for unconsciousness	97.8 (95.3, 99.0)
Screening for chest indrawing	97.1 (95.1, 98.3)
Screening for convulsions	97.9 (96.2, 98.9)
Screening for inability to breastfeed	98.2 (96.3, 99.2)
Screening for persistent vomits	98.2 (96.9, 99.0)
Screening for oedema	97.7 (95.3, 98.8)

**Table 4:** Performance score of CORP on malnutrition treatment procedures, accounting for clustering at CORP level

Procedure	Score (95% CI)
Ability to take MUAC measurement	96.5 (94.1, 97.9)
Ability to match MUAC colour on register	99.5 (98.6, 99.8)
Correct referral decision	99.8 (98.8, 100.0)
Achieved a conducive environment appetite test	96.5 (94.6, 97.7)
instructed caregiver to wash hands	97.0 (95.0, 98.2)
Conducted appetite test correctly (one-third of RUTF eaten)	99.8 (98.8, 100.0)
Made correct decision on appetite test	99.5 (98.5, 99.9)
Started weighing with scale re-calibration	99.5 (97.9, 99.9)
heavy pieces of clothing removed	98.4 (96.7, 99.2)
Calculated correct daily RUTF dosage	99.8 (98.9, 100.0)
Calculated correct number of RUTF for 1 week	99.2 (97.7, 99.7)
Recorded dosage on register correctly	99.5 (98.0, 99.9)
Correct amoxicillin prescription	94.4 (89.0, 97.2)
Recorded amoxicillin on register correctly	93.7 (87.6, 96.9)
Albendazole prescribed correctly	83.6 (72.9, 90.6)
Recorded albendazole on register correctly	82.2 (71.4, 89.5)
Ability to know treatment status (continue or stop)	100

**Table 5:** Performance score of CORP on malnutrition treatment messages to caregivers, accounting for clustering at CORP level

Procedure	Score (95% CI)
Gave adequate message to caregiver on RUTF feeding	90.4 (84.8, 93.6)
Gave adequate message to caregiver on ‘no sharing’ of RUTF	98.1 (96.0, 99.1)
Gave adequate message to caregiver on daily dosage of RUTF	99.7 (98.7, 99.9)
Gave adequate message to caregiver on next visit	98.7 (97.4, 99.4)

#### Treatment records

##### Demographic characteristics of enrolled children

Of the 303 children enrolled, complete records with all data elements were available for 288 children (95%). Information on child’s gender, mother’s age and level of education as well as other nutrition-related characteristics of the household are contained in Table 6.

**Table 6:** Background and demographic information of study enrolees

	N	Percentage
**Child’s sex**		
Male	152	52.8
Female	136	47.2
**Mother’s age (years)**		
< 20	9	3.1
20–29	148	51.4
30–39	106	36.8
40–49	20	6.9
50+	3	1.0
Do not know	2	0.7
**Mother’s level of education**		
None	196	68.1
Primary	14	4.9
Secondary	8	2.8
Other	70	24.2
**Child’s primary caregiver**		
Mother	246	85.4
Father	33	11.4
Grand mother	7	2.4
Sibling	1	0.4
Other	1	0.4
**Has the child ever breastfed?**		
Yes	255	88.5
No	32	11.1
Do not know	1	0.4
**Mother’s number of pregnancies**		
Median	4	
Mean	4.7	
Interquartile range (IQR)	2-7	
Range	1-5	
**Number of under-fives in the household**		
Median	2	
Mean	3.3	
IQR	2–3	
Range	0–18	
**Child’s age at enrolment (months)**		
Median	15	
Mean	17	
IQR	12–24	
Range	6–59	
**Child’s MUAC score at enrolment (cm)**		
Median	11	
Mean	10.8	
IQR	10.5–11.2	
Range	8–11.5	
**Child’s MUAC colour at enrolment**		
Dark Red: 58 (20%)		
Pink: 230 (80%)		

##### Treatment outcomes

Of the 303 children enrolled for treatment of SAM, treatment outcomes were available for 288 (96%). As Table 7 indicates, there was a cure rate of 73.5%, excluding referrals from the denominator in line with the standard method of calculating recovery rates in nutrition interventions. The median number of weeks in treatment for the cured children was 6.7. The most common reason for defaulting was that the caregiver did not wish to continue with the treatment or had sought alternative care elsewhere (36%). Forty-three (14.8%) were referred with over half (56%) of these cases being due to failed appetite test.

**Table 7:** Treatment outcomes

Outcome	^a^Excluding referred cases		Including referred cases		No of weeks in treatment
	*n* = 255	Percentage	*N* = 288	Percentage	
Cured	180	73.5	180	62.5	Median: 6 Mean: 6.7 IQR: 5–8 Range: 4–12
Non-response	11	4.5	11	3.8	12
Default	54	22	54	18.8	Median: 6 Mean: 6.8 IQR: 5–8 Range: 4–8
Referred	Not applicable	Not Applicable	43	14.8	Median: 3 Mean: 4.1 IQR: 2–6 Range: 2–10
Mortality	0	0	0	0	Not applicable

^a^Standard definition of recovery rates in nutrition programmes

Table 8 shows the adjusted risk ratios of factors affecting recovery. Children who started in the dark red MUAC zone had 31% (95% CI: 7–49%) less likelihood of recovery compared to those that started in the pink zone. Children seen by CORPs with catchment populations of 50 and above, up to less than 200, tend to have a lower chance of recovery compared to those with catchment population less than fifty. Maternal religion also found to impact recovery, which may be a proxy for socioeconomic or cultural drivers of recovery.

**Table 8:** Factors affecting treatment outcomes

	Adjusted risk ratio (ref: not recovered)
Dark red MUAC start (ref: pink MUAC)	0.69 (0.51, 0.93)
Age in months (continuous)	1.01 (1.00, 1.02)
# of under-five children in the house	1.00 (0.97, 1.03)
# of pregnancies of the mother	0.99 (0.95, 1.02)
Mothers age (in increment of 10)	1.08 (0.93, 1.25)
Maternal education (ref: no education)	
Primary Education	1.06 (0.79, 1.44)
Junior/senior secondary	0.94 (0.63, 1.39)
Other (e.g. Koranic education)	1.02 (0.81, 1.29)
Maternal religion (ref: Christian)	
Muslim	0.66 (0.48, 0.89)
Other	0.46 (0.27, 0.79)
Ever breastfed (ref: no)	0.82 (0.54, 1.24)
# of yrs. working as CORP	1.10 (0.93, 1.30)
Catchment size (ref < 50)	
50- < 100	0.70 (0.56, 0.88)
100- < 150	0.76 (0.58, 1.01)
150- < 200	0.68 (0.50, 0.91)
200+	0.75 (0.48, 1.16)
CORP religion (ref: Christian)	
Muslim	1.15 (0.82, 1.59)

### Qualitative results

#### Perceptions of key stakeholders on ability of CHWs to combine SAM and iCCM services

The FGD and IDI responses were categorised according to the following pre-determined themes: perception of malnutrition, general impression of the project, community-CORP relationship with regard to the treatment, workload of CORPs, supervision, supply chain, tools, referral mechanism and sustainability. Details of the qualitative data are contained in the supplementary file.

#### Acute malnutrition as a concept

Several caregiver FGD respondents reported that malnutrition is defined by a loss of weight or a “shrunken” look on a child, with a few mentioning that fever, by definition, is a part of malnutrition and a few others reporting that it is due to spiritual causes. It appeared from the responses that there was no clear distinction of malnutrition as a specific disease; the respondents generally described it as a state of weakness and illness. The two individuals who noted spiritual causes indicated that they realized that the illness was due to a different cause when their children eventually recovered after treatment. One IDI respondent blamed spiritual forces for her child’s failure to recover. Several respondents noted that the illness was a result of the mother getting pregnant while still breastfeeding, and others reported that, prior to receiving treatment, their families thought the illness was not curable and did not believe the treatment would work.

#### General impressions of the CORPs’ treatment program

All respondents expressed positive disposition toward the program indicating child’s recovery, free care, and shorter distance to access care as some positives. One supervisor mentioned that:

*“formerly, if there is a case of red MUAC, it is abandoned because there is no access to treatment.”*

A few caregivers reported that their family members were relieved upon hearing that there were no costs associated. One reported that her husband thought that treatment was expensive but upon notification that the treatment was free, he was very happy. Another reported that the respondent’s grandmother was already considering what personal belongings to sell in order to afford the treatment.

#### Community-CHW relationship surrounding treatment

In FGDs, CORP respondents reported being more respected by the community and that they are better recognized by the community members. A few of the CORPs also reported receiving gifts such as a chicken, grains, and money to fuel their motorcycles. Despite this expression of gratitude for the CORP treatment program, a frequently reported perception of the community was that the CORPs were biased or displayed favouritism in deciding who should or should not receive treatment. This issue was raised in both CORPs and caregiver groups where situations in which two children would be screened at the same time, one would be deemed eligible and the other not were described. This led to dissatisfied caregivers accusing CORPs of favouritism. A CORP mentioned that a caregiver felt it was her child’s right as a community member to be given RUTF while another noted that some community members were not aware that the service was meant only for malnourished children (Appendix 3 box 4).

#### Workload of CORPs

Supervisors and community health program managers all raised concerns about workload, and several of these respondents suggested the need to pay CORPs for the additional work. However, the CORPs themselves in their responses appeared to be happy with the addition of the nutrition treatment program to their tasks as this resulted in them gaining more respect from community members. Rather than requesting payment as a compensation for the extra workload, the CORPs expressed satisfaction and felt compensated by the knowledge and skill gained by treating malnourished children. During the intervention, CORP were requested to identify a convenient clinic day and time that worked with the schedules of their primary occupation (e.g. identify 1–2 days a week, limit to evening times). The CORPs stated that they did not feel burdened by the SAM treatment schedules because they were given the opportunity to choose convenient time for the treatment.

#### Supervision

The supervisors reported issues of caregivers not making timely visits or CORPs not being available at time of visiting, leaving situations in which either party is waiting for the other. Mobility for supervisors was also difficult due to long distances or bad roads. Frequency of supervision was brought up as a difficulty by program managers, given that for the iCCM program, the CORPs were only receiving quarterly supervision and the distribution of iCCM medication only happens every 2 months. Suggestions for improving supervision did not have an overarching theme, with suggestions ranging from making sure CORPs have all the tools, more financing, more manpower for supervision, having a calendar to ensure supervision visits are not missed, and reduced frequency of supervision (to monthly, quarterly). In FGDs, CORPs reported that having supervisors correct their mistakes in person was much appreciated. One respondent however indicated that the supervisors should not correct them in front of the caregivers, as it makes them look as if they do not know what they are doing (Appendix 3 box 6).

#### Supply chain

CORP supervisors appeared to have some difficulty in moving the RUTF around to hard-to-reach places, as by definition iCCM is prioritised for areas which are hard to reach. Programme implementers during IDI, indicated that initial procurement of RUTF was also very challenging, given that there was no existing community level nutrition programme in the area. Despite these difficulties, only one CORP FGD had a few respondents report stock-outs, while others indicated that they never had any issues with stock. One respondent indicated that the CORP supervisor would even borrow excess stock from others to ensure there was no stock-out. Another issue raised regarding keeping stock at home was ants being attracted by empty RUTF sachets.

#### Tools

The difficulty of calibrating the weighing scale was mentioned several times and doing MUAC measurement on an active child was also raised as an issue. A CORP suggested that a specific stand be designed for a weight scale rather than hanging it from a tree. A few people raised issues about how best to record data for individuals who do not regularly come, or in other words, how best to record a missed visit. Other suggestions included having an identification card for SAM children like they do for iCCM and uniforms for CORPs providing SAM treatment.

#### Referral mechanism

Referrals appeared to have been difficult, given that the care provided by the CORP was free and the referral would cost money. The referral site was approximately 80 km away from the intervention sites. Specifically, with appetite test failure, one CORP mentioned that a caregiver insisted that her child be enrolled to receive RUTF, despite the explanation that the child needed to be referred. Some also appeared to perceive this as an issue of favouritism; that certain CORPs did not want to treat them, hence they referred them to the health facility. Reasons brought up by CORPs supervisors for incomplete referrals included financial limitations, ignorance, families being convinced that the child is going to die anyway if CORP were not able to cure, and belief that the cause was spiritual.

#### Sustainability

Several respondents stressed the need for government ownership, and the need for results to be shared with local and village level leadership to receive buy-in and inspire collective responsibility. A few additionally suggested that the State Ministry of Health should identify a budget to invest in this program. In contrast, one respondent said that the program responsibility should remain with Malaria Consortium, as the local government does not have the capacity to sustain the program.

## Discussion

The study demonstrated that non-clinical CORPs could accurately follow the treatment protocol for managing SAM cases using simplified tools with adequate supervision and an intact supply system. The high competency scores seen in this study is similar to other studies in South Sudan and Bangladesh [12, 18]. although the scores were higher for the first assessment than recorded in South Sudan. ^(12)^ This could be linked to better educational status and experiences of the CORPs in iCCM in our study. The only relatively lower scoring task, albendazole prescription, was one of the tasks that was not part of the iCCM protocol. In addition, the incremental points in score per additional supervision showed that the excellent performance of the CORPs could also be due to the regularity of supervision provided as it has been found to increase performance of CORPs [12]. Supervision also builds confidence (through positive feedbacks) and improves self-efficacy [12]. However, the weekly frequency of supervision for the study may not be feasible at scale. Monthly supervision was most commonly suggested as more feasible, which is supported by the increasingly high performance of the CORPs over time. To maintain the observable quality outcomes of performance, confidence and self-efficacy attributable to supervision, an alternating site and meeting-like (group and scenario-based) monthly supportive supervision may be more sustainable as the burden of travels will be equally shared between the supervisors and supervisees [19].

The cure rate of the enrolled SAM cases was non-inferior to the minimum Sphere standard for treatment of SAM [20]. A 2016 assessment of health facility-based outpatient therapeutic programmes (OTPs) conducted in a similar setting in Nigeria revealed a much lower cure rate of 58% [20]. Similar study conducted in South Sudan documented a slightly higher recovery rate, this probably was due to the longer treatment period of 16 weeks compared to this study that discharged children at 12 weeks as non-response.^16^ The study’s mortality rate was also lower than Sphere’s recommended limit, potentially due to the relatively high referral rate by participating CORPs. These positive outcomes may be attributable to the simplicity of the protocol and tools, quality of training and supervision received by CHWs as well as early identification of cases before they deteriorated into more severe conditions.

Nonetheless, it is worth noting that supervisors suspected that CORPs were wrongly referring children enrolled on the dark red MUAC zone because of the perceived danger. The study’s default rate was higher compared to the Sphere standard. It is recognised that OTPs’ default rates can be very high, but all the factors that usually contribute to a high OTP default rate [21] (such as OTP centres being far from caregivers, caregivers having to tackle difficult terrain to reach such centres, and there being a poor and costly transportation network locally) were not present in the study sites. Indeed, caregivers who participated in FGDs specifically mentioned appreciating their proximity to treatment centres (i.e. CORPs’ houses). Furthermore, the fact that RUTF and CORPs’ services were free and SAM treatment highly desirable in caregivers’ eyes suggests that defaults were due to reasons unrelated to quality, access and cost of treatment (e.g. caregivers preferring to seek alternative, often spiritual, means of treatment). The high default rate was possibly due to malnutrition not recognised as a health problem requiring treatment among rural dwellers [22]. This was also corroborated by FGD and IDI participants where some of them believed that malnutrition needed spiritual solutions that cannot be provided by CORPs.

Findings from qualitative information indicated that acceptance was high among caregivers, CORPs and programme managers. CORPs felt motivated and happy due to the visible results of the treatment they were providing. The complaints of some caregivers that CORPs had been selective and discriminatory might be due to the fact that CORPs objectively determined whether children were eligible for SAM treatment according to the protocol, which caregivers were not familiar with. It might also be an indication that more effort needs to be made to inform the community about the nature of the programme and the use of RUTF as a medicine not a food.

There are a few limitations to bear in mind when interpreting the results of the study. Firstly, its scope did not include assessment of the effect of the extra requirements of SAM management on the quality of care of other diseases covered under the existing iCCM programme. A similar study in Bangladesh based on disease case scenario assessment of CHWs combining SAM treatment with curative and preventive services, found that quality of care might not necessarily be compromised [23]. However, a more practical assessment of curative services in form of clinical audit would give a better judgement of practice (skills and self-efficacy) rather than knowledge of treatment procedures obtainable in assessment by disease case scenarios. Also, a study within the Nigeria context will have better applicability in the country. Secondly, the study did not track the outcomes of the CORPs’ referrals and there was lack of resources to further investigate the causes of the high default rates observed. Demand creation activities were limited for the study. Further research is needed to fill these evidence gaps.

## Conclusion

The study demonstrated that with training and supportive supervision, CORPs in Nigeria can acquire the knowledge and skills required to assess, identify and treat SAM among under-fives, and refer cases using simplified protocols and tools, as part of an iCCM programme. This approach seemed acceptable to all stakeholders, however, further studies need to be commissioned to assess the effect of the extra workload on the quality of care provided by the CORPs with the integration of SAM and iCCM as well as determination of a viable supply chain system, cost effectiveness and coverage that could be achieved.

## Supplementary Information

**Additional file 1.**

## Data Availability

All datasets supporting the conclusions of this article is included within the article and supporting file.

## Abbreviations

CHEWs: Community Health Extension Workers
CHWs: Community Health Workers
CORPs: Community-Oriented Resource Persons
FGD: Focus Group Discussions
IDI: In-Depth Interviews
ICCM: Integrated Community Case Management of Childhood Illnesses
IRC: International Rescue Committee
MC: Malaria Consortium
MUAC: Mid-Upper Arm Circumference
MAM: Moderate Acute Malnutrition
NDHS: National Demographic and Health Survey
OTPS: Outpatient Therapeutic Programmes
RUTF: Ready-To-Use Therapeutic Food
SAM: Severe Acute Malnutrition
SHS: The Sphere Humanitarian Standard
