# Supplementary material for: A feasibility study to assess non-clinical community health workers’ capacity to use simplified protocols and tools to treat severe acute malnutrition in Niger state Nigeria
Source: BMC Health Serv Res. 2021 Oct 15;21:1102. doi: 10.1186/s12913-021-07118-4 (PMC8520247; doi:10.1186/s12913-021-07118-4)
Supplement: Supplementary file 1 — Additional file 1. [file 12913_2021_7118_MOESM1_ESM.docx]

**APPENDIX 1: Simplified protocol and tools**

**Box 1.** Mid-upper arm circumference MUAC tape

| 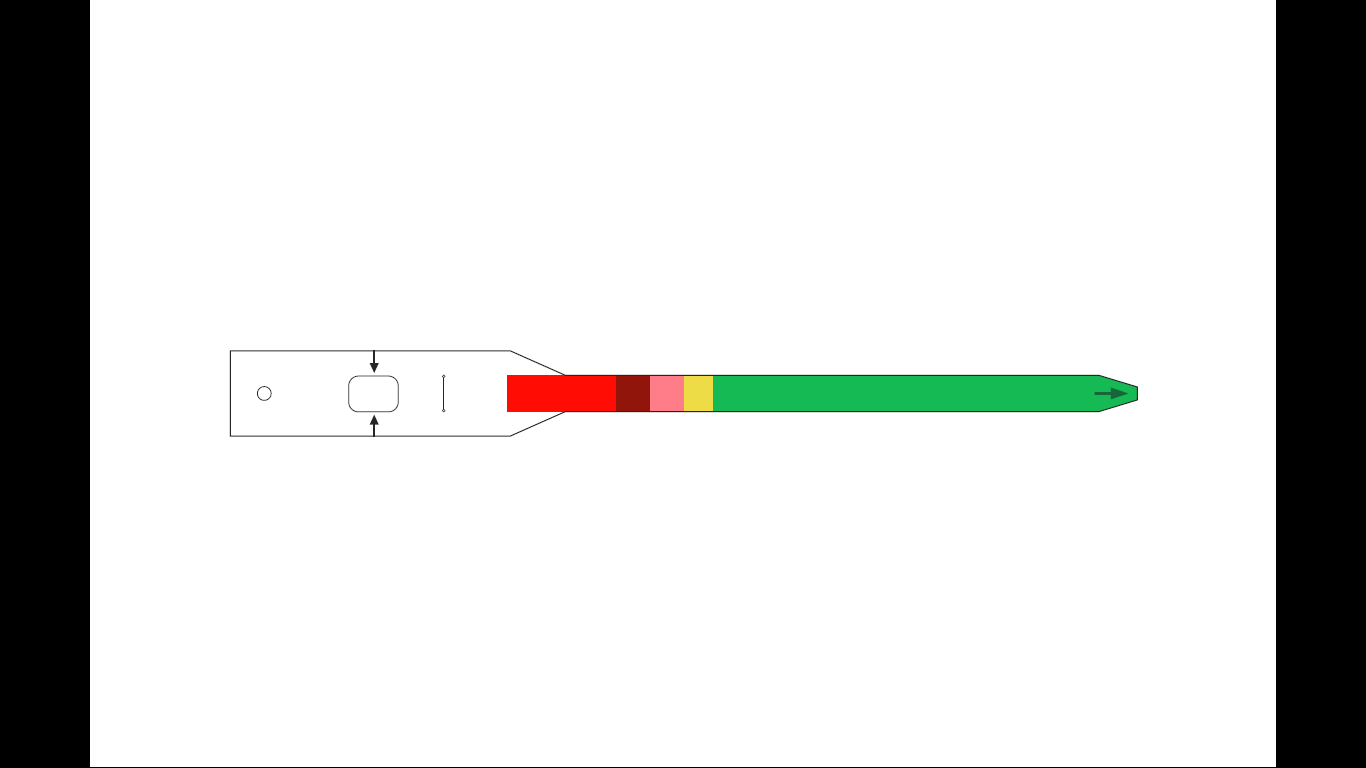  Figure 1: Mid-upper arm circumference tape   \| **Traditional tape** \| \| **Revised tape** \| \| \| --- \| --- \| --- \| --- \| \| **Categories** \| **Action** \| **Categories** \| **Action** \| \| **Red: <11.5cm** \| Treatment at OTP \| Red: <9cm \| Refer to nearest nutrition clinic – likely to need inpatient care \| \| Dark red: 9 - <10.25cm \| Treatment by CORP \| \| Pink: 10.25 - <11.5cm \| Treatment by CORP \| \| **Yellow: 11.5 to <12.5cm** \| Nutrition counselling \| Yellow: 11.5 - <12.5cm \| Nutrition counselling as per iCCM guidelines \| \| **Green - ≥12.5cm** \| No treatment \| Green - ≥12.5cm \| No treatment \| |
| --- | --- | --- | --- | --- | --- | --- | --- | --- | --- | --- | --- | --- | --- | --- | --- | --- | --- | --- | --- | --- | --- | --- | --- | --- |

**Box2: Simplified tools**

| 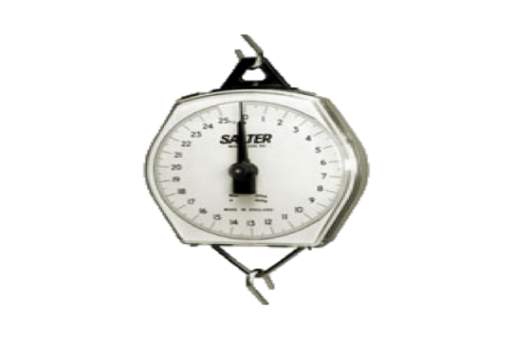 Ready-to-use therapeutic food (RUTF) dosage calculator  Ready-to-use therapeutic food (RUTF) dosage scale 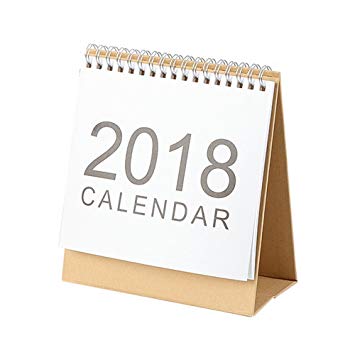 RUTF feeding message  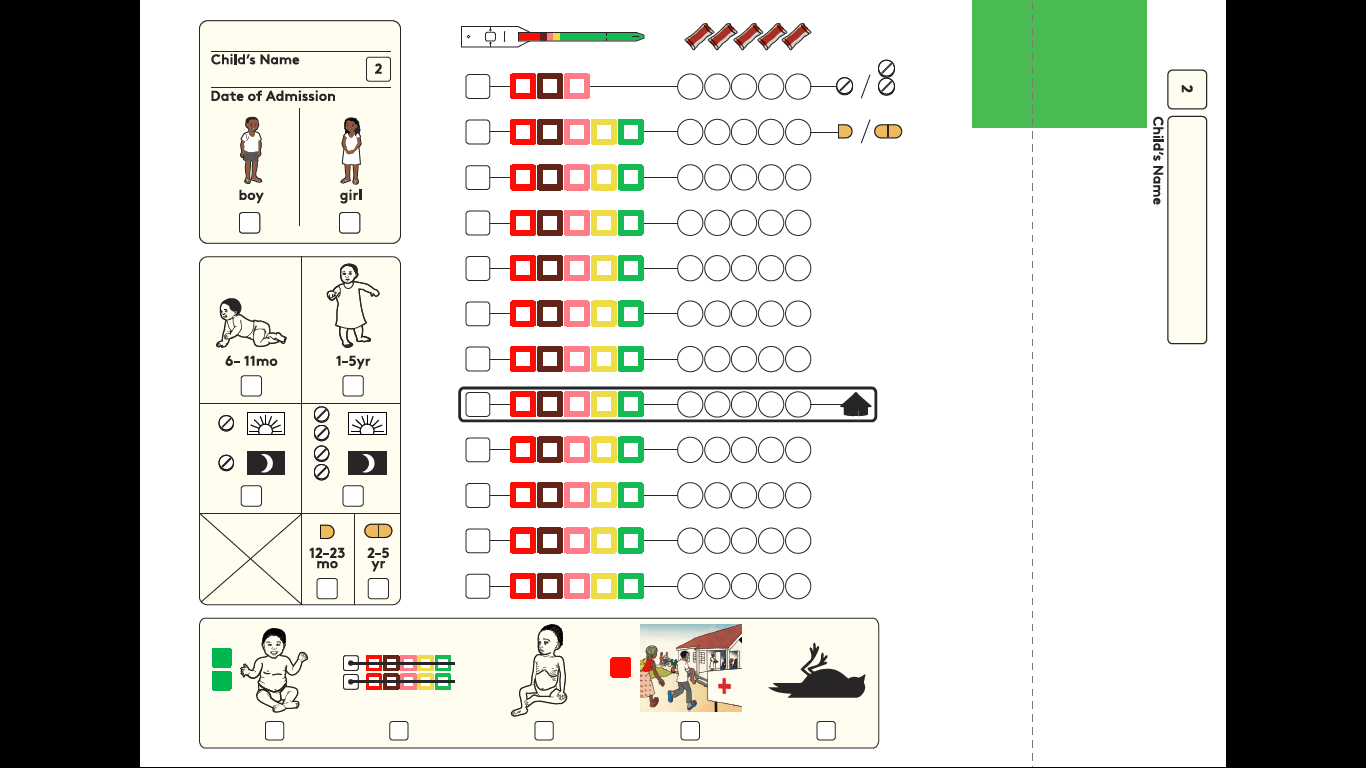  Patient register |
| --- |

**Box 3. Treatment Algorithm**

| 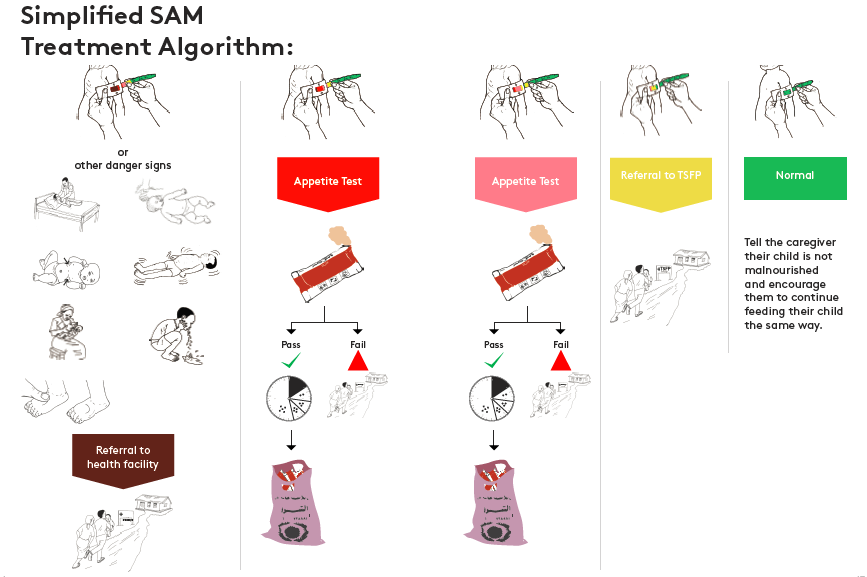 |
| --- |

**Appendix 2: Predictors of treatment response – recovery (single logistic regression**

| \|  \| Unadjusted risk ratio \| \| --- \| --- \| \| Dark red MUAC start (ref: pink MUAC) \| 0.71 (0.54, 0.95) \| \| Age in months (continuous) \| 1.01 (1.00, 1.02) \| \| # of under-five children in the house \| 1.00 (1.00, 1.01) \| \| # of pregnancies of the mother \| 1.00 (0.99, 1.01) \| \| Mothers age (in increment of 10) \| 1.06 (0.94, 1.19) \| \| Maternal education (ref: no education)  Primary Education  Junior/senior secondary  Other (e.g. Koranic education) \| 1.15 (0.81, 1.63)  1.21 (0.80, 1.83)  0.97 (0.78, 1.21) \| \| Religion (ref: Christian)  Muslim  Other \| 0.77 (0.60, 1.00)  0.54 (0.33, 0.89) \| \| Ever breastfed (ref: no) \| 1.05 (0.82, 1.35) \| \| # of yrs working as CORP \| 1.19 (1.04, 1.35) \| \| Catchment size (ref <50)  50-<100  100-<150  150-<200  200+ \| 0.70 (0.54, 0.90)  0.75 (0.58, 0.97)  0.70 (0.52, 0.94)  0.68 (0.46, 1.00) \| \| CORP religion (ref: Christian)  Muslim \| 1.22 (0.74, 2.00) \|  \| **Factors** \| **Adjusted risk ratio** \| \| --- \| --- \| \| Dark red MUAC start (ref: pink MUAC) \| 0.71 (0.53, 0.95) \| \| Caregiver education (ref: no education)  Primary Education  Junior/senior secondary  Other (e.g. Koranic education) \| 1.10 (0.80, 1.52)  0.91 (0.59, 1.42)  0.96 (0.76, 1.22) \| \| Caregiver religion (ref: Christian)  Muslim  Other \| 0.78 (0.57, 1.06)  0.55 (0.28, 1.07) \| \| # of yrs working as CORP \| 1.10 (0.96, 1.26) \| \| Catchment size  <50  50-<100  100-<150  150-<200  200+ \| Ref  0.69 (0.55, 0.87)  0.74 (0.60, 0.92)  0.69 (0.49, 0.97)  0.70 (0.45, 1.08) \| \| CORP religion  Christian  Muslim \| ref  1.15 (0.77, 1.71) \| |
| --- | --- | --- | --- | --- | --- | --- | --- | --- | --- | --- | --- | --- | --- | --- | --- | --- | --- | --- | --- | --- | --- | --- | --- | --- | --- | --- | --- | --- | --- | --- | --- | --- | --- | --- | --- | --- | --- | --- |

**APPENDIX 3.** **Quotes from in-depth interviews and focussed group discussions with frontline health workers, supervisors, caregivers and programme managers**

**Box 1.** **Qualitative data collection plan**

| \| **Participants** \| **Number of FGDs** \| **No of IDIs** \| **Selection procedure** \| \| --- \| --- \| --- \| --- \| \| CORPs \| 4 \| 4 \| **IDI:** Four CORPs (two highest performers and two lowest performers) based on assessment scores  **FGD:** Excluding CORPs who participated in IDIs, CORPs were grouped into 8 clusters out of which four were randomly selected and 8 CORPs randomly selected from each of the selected clusters \| \| CORPs supervisors \| 2 \| 0 \| Two groups of 8 supervisors each selected randomly per LGA \| \| Caregivers \| 2 \| 4 \| **IDI:** One caregiver randomly selected from the following treatment outcomes: cured, defaulted, non-response and referred  **FGD:** Excluding caregivers who participated in IDI, caregivers were grouped into 8 clusters out of which 2 clusters were randomly selected and 8 caregivers randomly selected from the 2 selected clusters \| \| Policy/decision makers \| 0 \| 3 \| Directors of Public/Primary Health care at LGA, State and National levels \| \| Programme Implementers \| 0 \| 6 \| Programme staff from NGOs and State Ministry of Health \| |
| --- | --- | --- | --- | --- | --- | --- | --- | --- | --- | --- | --- | --- | --- | --- | --- | --- | --- | --- | --- | --- | --- | --- | --- | --- |

**Box 2.** **Acute malnutrition as a concept**

| ***Caregivers’ perception of malnutrition***  *My understanding of malnutrition is when a child loses weight and the body is not looking good (Caregiver 5, Bangi, Mariga LGA)*  *it’s when a child’s body looks pale and shrunken (FGD Caregiver 3, Bangi, Mariga LGA)*  *It is when a child is always weak and looks fragile (FGD Caregiver 1, Bangi, Mariga LGA)*  *What I understand about malnutrition is that a child looks under age and will not be active (FGD Caregiver 2, Bobi, Mariga LGA)*  *My understanding about malnutrition is that the sick child always has fever (FGD Caregiver 6, Bobi, Mariga LGA)*  *The skin of a malnourished child looks shrunken (FGD Caregiver 1, Bobi, Mariga LGA)*  *What I understand about malnutrition is a child always falling sick due to spiritual attack (FGD Caregiver 1, T/Magajiya, Rijau LGA)*  *Malnutrition is when a child is always dull and looks weak (FGD Caregiver 3, T/Magajiya, Rijau LGA)*  *I understand malnutrition to be when a child always has consistent fever (FGD Caregiver 4, T/Magajiya, Rijau LGA)*  *My understanding of malnutrition is when a child loses weight (FGD Caregiver 1 Rijau, Rijau LGA)*  *It’s when a child is always sick and starts losing weight (FGD Caregiver 2 Rijau, Rijau LGA)*  *The understanding I have is when a child looks sick, has fever, looks emaciated and also like someone that is bewitched (FGD Caregiver 4 Rijau, Rijau LGA)* |
| --- |

**Box 3. General impressions of the CORP treatment program**

| **Caregiver positive impressions**  *We like everything (FGD All caregivers, Bangi, Mariga LGA)*  *I like the recovery I noticed in my child (FGD Caregiver 3, Bangi, Mariga LGA)*  *I like that my child is now looking healthy and fat (FGD Caregiver 1, Bangi, Mariga LGA)*  *The good thing is that my child is now able to sit and crawl (FGD Caregiver 1, Bangi, Mariga LGA)*  *The program should continue, we are happy and our children are getting better (FGD Caregiver 4, Bangi, Mariga LGA)*  *The CORP encouraged and followed up. This helped to improve the health condition of the child (FGD Caregiver 1, Bobi, Mariga LGA)*  *The drug is free and accessible (FGD Caregiver 2, Bobi, Mariga LGA)*  *R4. The CORP is in our community and he is treating children with malnutrition. My child also receives malnutrition treatment and he is now very active (FGD Caregiver 4, T/Magajiya, Rijau LGA)*  *The good thing about receiving malnutrition from the corps is that children (FDG Caregiver 1, T/Magajiya, Rijau LGA) on treatment respond fast and always looks plump.*  *Malnutrition treatment improves a child health (FGD Caregiver 3, T/Magajiya, Rijau LGA)*  *What I like is the help we receive by giving us this treatment. Truly speaking our children are now healthier (FGD Caregiver 1 Rijau, Rijau LGA)*  *The treatment is given to us free. Our children are healthy. You don’t have to transport yourself to another place and sometimes the CORPs come to our house (FGD Caregiver 6 Rijau, Rijau LGA)*  *What I like about the treatment is how my child recovered (Caregiver 1 Rijau, Rijau LGA)*  *The drug is free and as soon as a child with SAM case start taking it the child become heathy and looking plumpy (IDI Caregiver, Bobi, Mariga LGA)*  *My child became healthy when we commenced treatment (IDI Caregiver, Bangi, Mariga LGA)*  **Caregiver negative impressions**  *There was nothing bad about the treatment (FGD All caregivers, Bangi, Mariga LGA)*  *I don’t see any negative thing about receiving malnutrition treatment from the Corp. In fact they should continue providing treatment (FGD Caregiver 7, Bobi, Mariga LGA)*  *I don’t see any bad thing about the treatment because our children are healthy and plump (FGD Caregiver 4, Bobi, Mariga LGA)*  *I have not seen anything bad in receiving malnutrition treatment, because my child’s health improved while on malnutrition treatment (FGD Caregiver 1, T/Magajiya, Rijau LGA)*  *I see nothing bad about the treatment only good progress, regarding the health of my child (FGD Caregiver 3, T/Magajiya, Rijau LGA)*  *I didn’t find anything bad. My child is now ok (FGD Caregiver 2, T/Magajiya, Rijau LGA)*  *Nothing bad has happened since I began receiving malnutrition treatment (FGD Caregiver 4, T/Magajiya, Rijau LGA)*  *The other caregiver told me that the RUTF is effective but for me I did not see any thing good about the RUTF. The bad thing is that my child is unable to eat RUTF even if I give it to him ,he will put his hand into his mouth and remove the RUTF and throw it away (IDI, Rijau, Rijau LGA)*  **Impression of family members**  *They (family members) used to say that they thought the treatment will be expensive not knowing that it was even free (FGD Caregiver 3, Bangi, Mariga LGA)*  *Even me, my grandmother is already thinking of what to sale to pay for the treatment but we are happy it’s free (Caregiver 3, Bangi, Mariga LGA)*  *The family members thought it was a serious and different sickness (FGD Caregiver 3, Bobi, Mariga LGA)*  *Some were saying it was from the breast milk (FGD Caregiver 2, Bobi, Mariga LGA)*  *Some were suspecting it was as a result of pregnancy while still breast feeding (FGD Caregiver 1, Bobi, Mariga LGA)*  *R1. The member of my house hold thought that my child was suffering from fever and they always ask me to go for treatment at the hospital (FGD Caregiver 1, T/Magajiya, Rijau LGA)*  *They thought it was a sickness which I could use herbals (FGD Caregiver 2, T/Magajiya, Rijau LGA)*  *None of them thought it was malnutrition, because they don’t have the knowledge of malnutrition (FGD Caregiver 3, T/Magajiya, Rijau LGA)*  *My household thought that it was just a sickness as a result of breast feeding my child while being pregnant before weaning. They never knew it was malnutrition (FGD Caregiver 4, T/Magajiya, Rijau LGA)*  *My husband thought the treatment would be very expensive, when I told him that our child has been assessed and it was malnutrition, I told him the treatment was free and he was happy about it (FGD Caregiver 6, Rijau, Rijau LGA)*  *They never thought that the sickness is curable and that treatment will work (FGD Caregiver 1, Rijau, Rijau LGA)*  *They thought that the CORP will just waste his time, that the child is attacked spiritually (FGD Caregiver 5, Rijau, Rijau LGA)*  **Community perceptions**  *Community members think the treatment is good, I have never seen anyone who thinks badly about the program (FGD Caregiver 5, Bangi, Mariga LGA)*  *Community members think it’s a welcome one, since they see my husband going with me for treatment (FGD Caregiver 1, Bangi, Mariga LGA)*  *Community members felt happy (FGD Caregiver 3, Bobi, Mariga LGA)*  *Community members felt happy and also want their children to be enrolled (FGD Caregiver 7, Bobi, Mariga LGA)*  *The caregivers whose their children did not qualify felt the CORP is selective (FGD Caregiver 2, Bobi, Mariga LGA)*  *Caregivers felt the CORPs is biased and not transparent (FGD Caregiver 4, Bobi, Mariga LGA)*  *They see that my child’s health has improved and other caregivers in the community want the RUTF for their children (Caregiver 1, T/Magajiya, Rijau LGA)*  *Many caregivers want their children to be enrolled, seeing my child been active (FGD Caregiver 2, T/Magajiya, Rijau LGA)*  *It is a good program and it has improved children’s health in my community (Caregiver 4, T/Magajiya, Rijau LGA)*  *Every caregiver in my community is interested in in the program because they acknowledged the importance of malnutrition treatment there (FGD Caregiver 3, T/Magajiya, Rijau LGA)*  *Is a good program it stop our children from dying. Some of my relatives were surprised and asked me what I was giving to my child that is making him look good and plump. I told them that it’s the RUTF treatment that he is receiving (FGD Caregiver 6, Rijau, Rijau LGA)*  *They say it is wonderful, that children can now walk. My friends said that the drug is good that my child is looking good (FGD Caregiver 6, Rijau, Rijau LGA)*  *They think it’s a good treatment and are very happy about it treatment (Caregiver 1, T/Magajiya, Rijau LGA)*  *My community members felt it has come to stay and treat all SAM cases identified treatment (FGD Caregiver 2, T/Magajiya, Rijau LGA)*  *They welcome the program because it brought the treatment of SAM to their door step treatment (FGD Caregiver 3, T/Magajiya, Rijau LGA)*  *In my community they see it as a good development treatment (FGD Caregiver 4, T/Magajiya, Rijau LGA)*  **Difficulty in accessing service**  *No difficulty (FGD All caregivers, Bangi, Mariga LGA)*  *No problems even my husband used to carry me to collect RUTF from the corps (FGD Caregiver 3, Bangi, Mariga LGA)*  *No, I have never experienced any difficulty during treatment (FGD Caregiver 1, Bobi, Mariga LGA)*  *I have no problem, each time I visited the CORPs we receive treatment (FGD Caregiver 3, Bobi, Mariga LGA)*  *I did not have any experience or challenge with the CORP when receiving treatment (FGD Caregiver 1, T/Magajiya, Rijau LGA)*  *The corps always gives us RUTF whenever I go on my appointment day I had no problem with receiving malnutrition treatment (FGD Caregiver 2, T/Magajiya, Rijau LGA)*  *I have no problem regarding treatment from the CORP he always welcome me when I go (FGD Caregiver 3, T/Magajiya, Rijau LGA)*  *No difficulty (FGD Caregiver 1, Rijau, Rijau LGA)*  *No delay. Immediately we go to the CORP he will give us the treatment (FGD Caregiver 6, Rijau, Rijau LGA)*  **Difficulties in weekly visits for treatment**  *No difficulty (FGD Caregiver 5, Bangi, Mariga LGA)*  *No difficulty, On Monday we don’t go to anywhere because is clinic day (FGD Caregiver 1, Bangi, Mariga LGA)*  *I go back every week and my husband makes sure I go back (FGD Caregiver 1, Bobi, Mariga LGA)*  *I never experienced difficulties because my husband supports me to go back (FGD Caregiver 7, Bobi, Mariga LGA)*  *I never experienced challenges with going back (FGD Caregiver 4, Bobi, Mariga LGA)*  *I go back every week and the CORP attends to me (FGD Caregiver 5, Bobi, Mariga LGA)*  *I go back every week because my husband makes sure I go (FGD Caregiver 2, Bobi, Mariga LGA)*  *I go back every week (FGD Caregiver 3, Bobi, Mariga LGA)*  *My child suddenly refuses the RUTF that was the reason why I stopped going back (FGD Caregiver 2, T/Magajiya, Rijau LGA)*  *I always go back every week for the drug, I have never had any difficulties (FGD Caregiver 3, T/Magajiya, Rijau LGA)*  *I always go back to collect RUTF without any problem every week (FGD Caregiver 4, T/Magajiya, Rijau LGA)*  *I always go back every week even when my child was purging. I never experienced any difficulties (FGD Caregiver 1, T/Magajiya, Rijau LGA)*  *We don’t default and don’t have difficulty when the week comes we leave whatever we are doing and come for treatment (FGD All caregiver, Rijau, Rijau LGA)*  *I don’t have problem as such because I make sure I visit the CORP every week as scheduled due to the importance attached to it (IDI, Caregiver, Bobi, Mariga LGA)*  *When I realised that my child didn’t like the RUTF, I decide to return the ones I collected from the CORP after the 3rd week and since then I did not care to go back to him again (IDI, CG, Rijau, Rijau LGA)*  *There was a time i travelled for wedding and i missed a week (IDI, CG, T/Magajiya, Rijau LGA)*  **Difficulties in home treatment by caregivers**  *Initially my child doesn’t eat it but later he starts eating very well (FGD Caregiver 5, Bangi, Mariga LGA)*  *There were no difficulties, my child is always eager to eat the RUTF (FGD Caregiver 2, Bobi, Mariga LGA)*  *I never experienced difficulty giving my child RUTF (FGD Caregiver 4, Bobi, Mariga LGA)*  *My child like eating it (FGD Caregiver 6, Bobi, Mariga LGA)*  *I never experienced difficulty giving my child RUTF (Caregiver 5, Bobi, Mariga LGA)*  *I have not experienced difficulty giving my child RUTF (Caregiver 3, Bobi, Mariga LGA)*  *My child enjoys eating it (Caregiver 7, Bobi, Mariga LGA)*  *My child wants to eat the RUTF always (Caregiver 1, Bobi, Mariga LGA)*  *There were no difficulties, my child enjoyed eating the drug (Caregiver 3, T/Magajiya, Rijau LGA)*  *My child was purging when he was eating the RUTF that was the only difficulties I experienced (Caregiver 4, T/Magajiya, Rijau LGA)*  *The child always ate the RUTF and never wants it to finish that was the difficulty I had (Caregiver 2, T/Magajiya, Rijau LGA)*  *No difficulty because the child always eats it (Caregivers 4 & 6, Rijau, Rijau LGA)*  *When I start giving her RUTF the child refuse to eat so I complain to the CORP and he said I should not worry with time the child will become used to it and the child will eat the RUTF (IDI, CG, Bobi, Mariga LGA)*  *my child rejected the RUTF totally and even when the CORP visited me , he advice me to come back and I refuse because my child will not take the drugs and I don’t want to waste them (IDI, CG, Rijau, Rijau LGA)*  *The RUTF given to him for a week is never enough for him in a week. I have to borrow from my colleague that her child is also on treatment. Her child does not finish his dosage (IDI, CG, T/Magajiya, Rijau LGA)*  **Effect on the project on feeding other children**  *No effect, the children still eat their normal food (All caregivers, Bangi, Mariga LGA)*  *It has affected it because I try to give them good food whenever I am to give the malnourished child RUTF (Caregiver 3, Bobi, Mariga LGA)*  *I prepare their food very well now (Caregiver 4, Bobi, Mariga LGA)*  *They eat at the same time I am to feed the malnourished child with RUTF (Caregiver 7, Bobi, Mariga LGA)*  *Since the RUTF is easy to give, after giving it; I will cook for other children, including the sick child, so nothing changes (Caregiver 6, Rijau, Rijau LGA)*  *It doesn’t affect how I feed other children in my house but other children in the house also cries to eat RUTF always (Caregiver 4, T/Magajiya, Rijau LGA)*  *Other children eat their own food, I find it difficult to feed the malnourished child with home food. Because he is used to RUTF (Caregiver 1, T/Magajiya, Rijau LGA)*  *Yes. My child is used to RUTF but other children at home eat whatever I cook at home so it has no effect on our home feeding (Caregiver 2, T/Magajiya, Rijau LGA)*  **Suggestions on improvement of community treatment of malnutrition**  *Continuity of the program and every child should be considered (Caregiver 1, Bobi, Mariga LGA)*  *Increase supply of RUTF and continuity of the program (Caregiver 2, Bobi, Mariga LGA)*  *RUTF should be given throughout the year for all malnourished children that qualify for treatment (Caregiver 2, T/Magajiya, Rijau LGA)*  *RUTF should be given to all malnourished children in our community to improve their health (Caregiver 1, T/Magajiya, Rijau LGA)*  *The CORP shouldn’t stop giving RUTF to children on malnutrition treatment until that child has finished growing (Caregiver 4, T/Magajiya, Rijau LGA)*  *The treatment should continue, because it has helped children with malnutrition in our community (Caregiver 3, T/Magajiya, Rijau LGA)*  *Financial support to motivate the CORPs for example some CORPs are being transferred from a different community to help other communities who do not have people that can read or write (Supervisor 2, Mariga LGA)*  **CORPs’ impression about community-level treatment of malnourishment**  *In my own opinion I think it is a good development having malnutrition program in my community because malnourished children are been treated within the community and the caregiver of the malnourished children are very happy (CORP 2, T/Magajiya, Rijau LGA)*  *Members of my community wish for continuity of the malnutrition program because of the treatment outcome of the malnourished children they witness (CORP 1, T/Magajiya, Rijau LGA)*  *I think it’s a good development for my community. Caregivers of malnourished children in my community travel to Kebbi state to seek for malnutrition treatment before it began in my community. But now they access malnutrition treatment in the community without undergoing the stress of travelling to Kebbi state for treatment (CORP 3, T/Magajiya, Rijau LGA)*  *My community members are very happy to have malnutrition treatment for the malnourished children in our own community (CORP 4, T/Magajiya, Rijau LGA)*  *I think the programme is a good one, with the programme in my community people are happy and wish that the program continue (CORP 1, Bangi, Mariga LGA)*  *I think the programme opened my eyes because I never knew that we had malnourished children in our community until when I started malnutrition treatment. People are happy in my community and wishes that the program continues (CORP 2, Bangi, Mariga LGA)*  *It’s a welcome idea, because the treatment is free and we have malnourished children in my community they don’t need to pay for the treatment (CORP 3, Bangi, Mariga LGA)*  *I think it is a great programme, in my community all malnourished cases are usually treated in Kebbi state but now it’s been treated by me in the community. They don’t have to travel to Kebbi for malnutrition treatment again (CORP 4, Bangi, Mariga LGA)*  *It’s very good and community members are very happy when they heard about the drugs because caregivers travel to Kebbi states for the treatment of SAM, thank God the drugs are now here free and safe for consumption (CORP 1, Rijau, Rijau LGA)*  *The programme is very effective. Through this program we are able to identify cases of malnutrition most especially the children on pink colour within three weeks of visit the child starts changing from yellow to green which shows that the treatment is very effective and it gives the less privilege children hope for life (CORP 2, Rijau, Rijau LGA)*  *The community member are very happy about the treatment (CORP 2, Rijau, Rijau LGA)*  *The community members said the drug is 100% effective and pray for its continuity (CORP 3, Rijau, Rijau LGA)*  *The community members think the treatment is okay, because some of the children that were not walking before the treatment started walking and playing around with other children after the treatment (CORP 4, Rijau, Rijau LGA)*  **CORPs’ positive experience in the treatment**  *I like the fact that my community members don’t have to travel to Kebbi state to get malnutrition treatment again (CORP 1, T/Magajiya, Rijau LGA)*  *I like the method of administering RUTF to malnourished children and how they recovered (CORP 3, T/Magajiya, Rijau LGA)*  *The number of caregivers in my community that come to access malnutrition treatment is what I appreciate. At the end of the treatment you will see them cured and healthy (CORP 4, T/Magajiya, Rijau LGA)*  *I appreciate how my community members support me with some money to buy fuel in my motorcycle for transportation (CORP 1, Bangi, Mariga LGA)*  *What I like about malnutrition is the fact that children are not dying of malnutrition in my community again (CORP 3, Bangi, Mariga LGA)*  *Truly, I like everything about SAM treatment, I have visited many traditional medicine men with my nephew but they cannot treat SAM case, they will tell you it’s spiritual and I will still pay for the little they give me. Now that SAM treatment is available, whenever RUTF is given to any child with malnutrition, he becomes fresh and healthy and no money is paid for the treatment. Lastly it added more knowledge to us as a CORP (CORP 2, Rijau, Rijau LGA)*  *I thank God for being one of the participant, what I like are the tools, they are simple to use (CORP 5, Rijau, Rijau LGA)*  *What I like is the effectiveness of the drugs. (CORP 3, Rijau, Rijau LGA)*  **CORPs’ dislikes about the *treatment***  *Some caregivers don’t come on time on the day of their appointment (CORP 3, T/Magajiya, Rijau LGA)*  *Some children used to fail appetite test and the caregivers don’t want to go for referral. They complain of money (CORP 4, T/Magajiya, Rijau LGA)*  *Some caregivers feel we don’t want to help them, if we refer them to the hospital. Because our treatment is free (CORP 1, T/Magajiya, Rijau LGA)*  *I face challenges with caregivers that come with under age children; they always want me to enrol their children for treatment even if I explain to them that the child does not qualify (CORP 2, Bangi, Mariga LGA)*  *Some caregivers felt that I am selective because their children didn’t qualify for malnutrition treatment and I didn’t enrol them (CORP 3, Bangi, Mariga LGA)*  *What makes the treatment difficult are (1) timer 2) weighing scale, I don’t have weighing scale, because I collect from nearby community. Weighing scale is one of the important tools for SAM because it determines the dosage for a child; while timer is used for counting breath per minute during the process of feeling sick child recording form my timer is not functioning (CORP 3, Rijau, Rijau LGA)*  *Caregivers that their children fail appetite test will still insist you must give them RUTF, even when you explain to them, they will not agree (CORP 2, Rijau, Rijau LGA)*  **CORPs’ supervisors’ positive impression about the project**  *From the Training we were told it’s a research. The programme is very good and very important, because it helped treat malnourished children and improve the health standard of the community of the community, and on the path of the supervisors they acquired more skills on the recent standards on treating SAM cases and on the path of the CORPs they acquired more knowledge in the treatment process. When some of the children were cured, it encouraged other caregivers to bring their children (Supervisor 4, Mariga LGA)*  *The programme is fantastic because it is a new experience for the CORPs and supervisors. It has helped to improve the lives of children in the community. Formerly they go to Kebbi state for treatment but now it is in Niger state, Mariga LGA. Caregivers are enjoying it especially when the child is cured after few weeks of treatment. One of the caregivers gave me soap and klin to show appreciation (Supervisor 3, Mariga LGA)*  *The SAM treatment is helpful to our communities, formerly if there is a case of red MUAC it is abandoned because there is no access to the treatment but now there is a cure for the children and they have recovered (Supervisor 1, Mariga LGA)*  *The SAM treatment has brought changes because formerly there were problems but now there are improvement in the communities, children with cases of malnutrition have been cured (Supervisor 2, Mariga LGA)*  *Caregivers know that the program for their children to be treated so they are very happy, they are enjoying it. So many caregivers say good things about the treatment. Caregivers see it as very important and essential treatment. The CORPs enjoyed the programme because the showed great concern for the malnourished children, welcoming the caregivers warmly, CORPs showed great interest in the treatment process, they enjoyed and appreciate the programme. In most communities huts or houses were built for CORPs because of high regards accorded them in the treatment of SAM. Community saw the RUTF as a great medicine because it cures their children so they hold the programme in high regards. The community member thanked the supervisors and CORPs for the programme (Supervisor 1, Mariga LGA)*  *The programme is a happy one. It helps to reduce infant death, improve their health status, it helps malnourished children, it helps to know the hard-to-reach areas and bring the treatment at their door steps (Supervisor 1, Rijau LGA)*  **CORPs’ supervisors’ negative impression about the project**  *The programme is a voluntary one, lack of funding is a challenge, the CORPs are working, no support, no motivation from the community and the caregivers because they feel that the corps are working for the government. The caregivers see it as a problem to keep coming for the treatment because of the duration (Supervisor 4, Mariga LGA)*  *Lack of motivation and support in monetary terms and encouragement (Supervisor 1, Mariga LGA)*  *Lack of financial support. (Supervisor 3, Mariga LGA)*  *Work load and motivation in form of money. The corps carrying out the treatment would have too much work and they are doing a voluntary work. If they are not supported financially it can be a challenge as they won’t be motivated to do the work well (Supervisor 2, Rijau LGA)*  **Programme Managers’ positive impressions on the project**  ***I****t is unquantifiable, some children that initially seemed terminal became healthy again as a result of the treatment (Trainer, SMoH)*  *The community appreciates and accepts (PHC Director)*  *I saw the treatment being brought to the doorstep in Hard-to-reach areas and it was helpful to reduce mortality, revive lives and reduce poverty (PHC Director)*  *It brings to bear the problem of malnutrition. Based on statistics done early 2017, Mariga LGA reported 7 cases of SAM, then the program started, we recorded almost over hundred we targeted 180 children with SAM but we treated over 300 and that proves great success in the treatment of malnutrition. the community and the community leaders are also impressed. so these are some highlights of positive things (NGO, PM)*  *The corps are competent, the data is impressive and the number of children been cured were quite encouraging from my end (SMOH, PM)*  *one of the positive things was that we were able to harvest children with malnutrition and those that were treated, it shows that if corps are equipped they will be able to treat malnutrition in the community (NGO, PM)*  *Irrespective of literacy rate the corps were able to use their basic knowledge to treat children, implement treatment outline and document treatment outcome. So it is quite encouraging. There was commitment on the part of the program manager, supervisors and community members and acceptance too (Clinician, Hospital)*  *The level of Trainings is a positive one I have seen, CORPs involvement, their willingness and enthusiasm, assigning supervisors for the CORPs to monitor them, little incentives for the corps is something that is appreciated. The SAM cases have been managed properly and the people are happy they have gotten solution to their probems and they welcomed it (PM, SMOH)*  **Programme Managers’ negative impressions on the project**  *The negative things were that the caregivers sometimes meet the corps with inadequate RUTF, so it is usually a different thing for the caregivers (PHC Director)*  *The SAM treatment selected only few communities and also the issue of ending the research study in the communities are some of the negative things I saw about the programme (PHC Director)*  *probably looking at the catchment area and the number of Corps we are using, it is not every community that has these CORPs and this project. There are communities that actually need these services which they cannot have, some seem to be very far from where this project is. Some have to trek to where these CORPs managing SAM cases are. It is a negative one. Another one is that there is no proper supervision from the state level (PM, SMOH)* |
| --- |

**Box 4.** **Community-CORP relationship surrounding treatment**

| ***How CORPs think caregivers perceive the project***  *The care givers saw it as a good program. When their children are treated they recover and become healthy (CORP 4, T/Magajiya, Rijau LGA)*  *It’s a good program, because it treats and cures SAM (CORP 2, T/Magajiya, Rijau LGA)*  *They think it’s a welcome programme and they are interested in the SAM program (CORP 1, T/Magajiya, Rijau LGA)*  *They are happy with SAM treatment support they receive (CORP 3, T/Magajiya, Rijau LGA)*  *They felt the programme is a gift given to their community (CORP 4, Bangi, Mariga LGA)*  *The care givers are glad and that the program is an act of sympathy for their community children with malnutrition problem (CORP 4, Bangi, Mariga LGA)*  *The caregiver are very happy about the program, because the health status of their children has improved (CORPs 2, 3 & 4, Rijau, Rijau LGA)*  ***Experience of CORPs with caregivers whose children were not qualified for enrolment into the project***  *The care givers used to be angry with me if their children did not qualify for SAM treatment (CORP 3, T/Magajiya, Rijau LGA)*  *They don’t used to be happy if their children did not qualify for SAM treatment (CORP 4, T/Magajiya, Rijau LGA)*  *They felt we are biased if their children did not qualify for SAM treatment (CORP 1, T/Magajiya, Rijau LGA)*  *The care givers usually feel we are just being selective, choosing those children we feel like treating for SAM (CORP 2, T/Magajiya, Rijau LGA)*  *The challenge I experienced was that some of the children brought to me for malnutrition treatment failed appetite test and their parents would take them home even if they have been referred and is not proper because they complained of lack of fund for hospital bills (CORP 1, Bangi, Mariga LGA)*  *I faced difficulties with caregivers who default while on treatment. The defaulted caregiver felt it is their right as a member of the community to be given RUTF at any time of their visit (CORP 2, Bangi, Mariga LGA)*  *I don’t have any difficulties with the caregiver because I explain to them all the protocols involved in the treatment (CORP 3, Bangi, Mariga LGA)*  *The caregiver are not always happy when you tell them their children are not eligible for SAM treatment such as those on green, yellow, red on MUAC and even the underage/ over age, including those that fail appetite test, they continue to disturb you that you must give them RUTF, sometimes the feel you are being biased (CORP 5, Rijau, Rijau LGA)*  ***Experience of CORPs with caregivers whose children were referred to hospital for further treatment***  *The caregivers never want to go for the referral, we have to persuade them before they agree (CORP 3, T/Magajiya, Rijau LGA)*  *The caregivers always plead to stay with me for the treatment but I advise them and they adhere to referral (CORP 4, T/Magajiya, Rijau LGA)*  *They don’t want to go because they feel we don’t want to help them. (Selective) so I follow to the hospital (CORP 1, T/Magajiya, Rijau LGA)*  *I explained to them the reason why I’m referring their children to the hospital and that is why they always accept the referral (CORP 1, Bangi, Mariga LGA)*  *I make the caregiver understand why I am referring them and also tell the caregiver that they will get good attention and treatment at their arrival at the hospital (CORP 4, Bangi, Mariga LGA)*  *I referred a case but she refused to go due to financial issues. I went to her house and encouraged her on the importance of adhering to referral (CORP 2, Rijau, Rijau LGA)*  *The child failed appetite test and I referred her. I went to her house and gave her the necessary advice on the importance of referral (CORP 2, Rijau, Rijau LGA)*  **Experience of CORPs with caregivers whose children were cured and discharged**  *The caregiver doesn’t want me to stop giving them RUTF. I have to explain, pet and advise them on what to do before they stop coming (CORP 1, T/Magajiya, Rijau LGA)*  *The caregiver complained that they are already used to the RUTF. I advised them on home food (CORP 2, T/Magajiya, Rijau LGA)*  *The difficulties I experienced are that the caregivers want to continue collecting RUTF from me even after discharge (CORP 4, Bangi, Mariga LGA)*  *Even if I explain and show them that the child is cured they will still be pleading to continue with the treatment because they can see that their children are looking fresh (CORP 6, Bangi, Mariga LGA)*  *They sometime plead to pay some money for the RUTF (CORP 1, Bangi, Mariga LGA)*  *Some caregivers want to buy the RUTF from me, so as to continue feeding their children with it because they felt it will affect the health of their children if they didn’t continue feeding their children with the RUTF (CORP 2, Bangi, Mariga LGA)*  *Some caregivers want to pay for the RUTF until when we refuse to sell it to them (CORP 3, Bangi, Mariga LGA)*  **Experience of CORPs with caregivers who default in weekly visits**  *I don’t have such cases in my community (CORP 1, T/Magajiya, Rijau LGA)*  *I experienced it with a caregiver in my community that travelled for a ceremony (CORP 2, T/Magajiya, Rijau LGA)*  *I experienced it with a caregiver who said another child was sick (CORP 3, T/Magajiya, Rijau LGA)*  *I have never experienced such case in my community (CORP 4, T/Magajiya, Rijau LGA)*  *Financial issues and lack of transportation prevent them (caregivers) from coming back (CORP 4, Rijau, Rijau LGA)*  **How caregivers were encouraged not to default**  *I advised them on the importance of their child’s health, they can come for treatment before going for any occasion always (CORP 2, T/Magajiya, Rijau LGA)*  *I always do follow up (CORP 3, T/Magajiya, Rijau LGA)*  *Some caregivers come back while other don’t because they are busy and forget. So I do follow up (CORP 1, Bangi, Mariga LGA)*  *In my community some caregivers feel that the child has recovered so no need to go back again. I usually advise them on the importance of coming back (CORP 2, Bangi, Mariga LGA)*  *Some caregivers failed to come due transportation and excuse of ceremony. I go for follow up (CORP 4, Bangi, Mariga LGA)*  *When they go for ceremony, they don’t come back on the appointed date. I always go to their houses to remind them (CORP 3, Bangi, Mariga LGA)*  *I tried to explain to the caregiver that if they are able to come back weekly it will determine the recovery of her child (CORP 4, Rijau, Rijau LGA)*  **Effect of project on CORPs’ relationship with community members**  *I am respected and honoured in my community because of SAM treatment (CORP 1, T/Magajiya, Rijau LGA)*  *I am being recognised more in my community due to SAM treatment (CORP 2, T/Magajiya, Rijau LGA)*  *People greet me even if I did not see them since I began SAM treatment (CORP 3, T/Magajiya, Rijau LGA)*  *I am being respected in my community since the beginning of SAM treatment (CORP 4, T/Magajiya, Rijau LGA)*  *Yes, I am being respected more in my community (CORP 6, Bangi, Mariga LGA)*  *I am being recognized more than before by the members of my community (CORP 3, Bangi, Mariga LGA)*  *People honour me in my community more than before (CORP 4, Bangi, Mariga LGA)*  *I’m now close to our community leader more than before (CORP 2, Bangi, Mariga LGA)*  *Some people look at me as an influential man in the community because of the visits I receive (CORP 1, Bangi, Mariga LGA)* |
| --- |

**Box 5.** **Workload of CORPs**

| **Ease of combining primary occupation with CORPs functions**  *Yes, it is easy with the clinical day it makes my work easier, because I do only SAM treatment on that day (CORP 1, T/Magajiya, Rijau LGA)*  *It is easy I dedicated a day for clinical and it’s only for SAM treatment. (CORP 2, T/Magajiya, Rijau LGA)*  *No problem I suspend every work on the clinical day, to attend to SAM patients (CORP 3, T/Magajiya, Rijau LGA)*  *Yes, it is easy I sacrifice clinical day for SAM treatment alone (CORP 4, T/Magajiya, Rijau LGA)*  *It is easy for me because I have a clinical day for treating malnutrition (CORP 1, Bangi, Mariga LGA)*  *I find it easy because I sacrifice a whole day for malnutrition treatment (CORP 3, Bangi, Mariga LGA)*  *My other house hold and business activities didn’t affect treatment of malnutrition that I offered because I have a schedule day for the treatment of malnutrition (CORP 2, Bangi, Mariga LGA)*  *We are told from the beginning that the work will need much of our time and availability, which we agreed to sacrifice so I planned on how to do my activities without problem (CORP 1, Bangi, Mariga LGA)*  *I informed all the caregivers of malnourished children that my treatment day is on Tuesday every week so it was easy for me (CORP 5, Bangi, Mariga LGA)*  *I have clinical day which makes the work easy (CORP 6, Bangi, Mariga LGA)*  *it is very easy because in a week only one day is kept aside for SAM treatment and other days in the week we are free (CORP 1, Rijau, Rijau LGA)*  **Feeling about combining iCCM with malnutrition treatment**  *I felt honoured to be selected among other CORP so I don’t think of the workload (CORP 1, T/Magajiya, Rijau LGA)*  *At first, I was scared of the workload and thinking it will be difficult but am happy to be a part of it (CORP 2, T/Magajiya, Rijau LGA)*  *I felt I couldn’t do it at the beginning of the training, but it has added more knowledge to me (CORP 3, T/Magajiya, Rijau LGA)*  *What came to my mind is that, I have not finished with ICCM how am I going to handle SAM but I found it easier handling both (CORP 4, T/Magajiya, Rijau LGA)*  *Treating malnutrition and ICCM at the same time, I felt it’s a development for me (CORP 1, Bangi, Mariga LGA)*  *I felt I will have more experience by treating malnutrition in addition to ICCM (CORP 2, Bangi, Mariga LGA)*  *I was afraid that I can’t treat malnutrition at the beginning but I feel like an expert now (CORP 3, Bangi, Mariga LGA)*  *I didn’t feel anything before and after being invited for malnutrition training because I felt I can deliver the services (CORP 4, Bangi, Mariga LGA)*  *I feel great because people will recognise me for offering malnutrition treatment in the community in addition to existing ICCM treatment that am doing (CORP 5, Bangi, Mariga LGA)*  *I felt happy for having additional work to the ICCM that am doing because it added more knowledge to me (CORP 6, Bangi, Mariga LGA)*  *The corps were been worked out treating ICCM and Malnutrition, spending much time treating one child and other caregivers waiting, so they had much work load (PM, SMOH)* |
| --- |

**Box 6.** **Supervision**

| **CORPs’ likes about supervision received**  *I like how the supervisor corrects me when I am conducting treatment (CORP 4, T/Magajiya, Rijau LGA)*  *I like how the supervisor works along with me to conduct SAM treatment during supervision (CORP 2, T/Magajiya, Rijau LGA)*  *I like how the supervisor conducts RUTF stock inventory with me during supervision (CORP 3, T/Magajiya, Rijau LGA)*  *I appreciate how my supervisor conducts refresher training for me when he comes for supervision (CORP 1, T/Magajiya, Rijau LGA)*  *I like how the supervisor used to correct me (CORP 1, Bangi, Mariga LGA)*  *I acquired more knowledge on the job (CORP 2, Bangi, Mariga LGA)*  *My supervisor used to remind me anything I forget (CORP 3, Bangi, Mariga LGA)*  *I learnt how to take stock of the balance of RUTF that remains (CORP 4, Bangi, Mariga LGA)*  *I like the way my supervisor corrects me when he comes for supervision and am conducting treatment on a malnourished child (CORP 5, Bangi, Mariga LGA)*  *The presence of my supervisor in the community during supervision makes the community member to value the treatment that I offer to their children (CORP 6, Bangi, Mariga LGA)*  *My supervisor corrects me when I make mistakes while conducting malnutrition treatment in his presence (CORP 7, Bangi, Mariga LGA)*  *The supervisor taught us and corrected our mistakes (CORP 1, Rijau, Rijau LGA)*  *When my supervisor is around it boosts my morale and the caregiver agrees on anything I tell them to do (CORP 2, Rijau, Rijau LGA)*  **CORPs’ suggestions on how to improve supervision**  *Let the present supervision method be maintained (CORP 2, T/Magajiya, Rijau LGA)*  *If the supervision continues this way it will be better (CORP 3, T/Magajiya, Rijau LGA)*  *The supervisor should always come along with all required SAM tool during supervision visit (CORP 4, T/Magajiya, Rijau LGA)*  *The program should provide motorcycle for the supervisors. This will make them to visit us every supervision period without excuse (CORP 4, Bangi, Mariga LGA)*  *Supervisors should be supported financially to visit us for supervision every week (CORP 7, Bangi, Mariga LGA)*  *Encouragement to the supervisors to always supervise us (CORP 5, Bangi, Mariga LGA)*  *Let the supervisors be motivated very well with training and logistics supports for supervision (CORP 3, Bangi, Mariga LGA)*  **Supervisors’ impression on challenges of supervision**  *Time wasting; some of the caregivers do not come on time so we have to wait for them. Far distance from one ward to another ward for supervision.*  *Not being mobile was another challenge. Unavailability of data tools, sometimes the supervisors usually go themselves to get these tools and even use their money to make photocopies of the tools e.g. checklist. Too frequent weekly visit to CORPs (Supervisor 4, Mariga LGA)*  *No financial support for the supervisors (Supervisor 1, Mariga LGA)*  *There are Hard-to-reach areas and high risk places so support in monetary form to maintain regularity for supervision. Constant supervision because of the nature of the SAM treatment, it requires close supervision (Supervisor 2, Mariga LGA)*  *Caregivers do not report on time because of remote areas where the live (Supervisor 1, Mariga LGA)*  *The CORPs is sometimes absent keeping the caregivers waiting and sometimes the caregivers would be absent and the corps would be waiting (Supervisor 1, Rijau LGA)*  *The supervisors supervising more than one corps is a challenge, because they Corps are in different locations (Supervisor 7, Rijau LGA)*  *Terrain is a challenge, the roads are bad (Supervisor 2, Rijau LGA)*  *The number of times you have to supervise and the duration of the treatment (Supervisor 5, Rijau LGA)*  **Perceived risks of no supervision**  *CORPs would not carry out their work well, they wont give the caregivers adequate information before or during the treatment. For example, washing of hands during appetite test. RUTF would not be administered properly which can endanger the life of the child (Supervisor 4, Mariga LGA)*  *If there is no good supervision, there would be poor treatment and poor performance on the path of the CORPs (Supervisor 1, Mariga LGA)*  *There would be no respect for the CORPs from the caregivers. So the supervision would let the caregivers know they are following orders (Supervisor 3, Mariga LGA)*  *There would be failure in the programme and the corps would do whatever they want to do (Supervisor 1, Rijau LGA)*  *There may be wrong readings in MUAC which may lead to improper dosage for the child (Supervisor 2, Mariga LGA)*  *Proper prescription may not be given, a case where there is danger sign under dose or over dose may be given (Supervisor 6, Mariga LGA)*  *There may be bad report from caregivers or community members about corps or vice versa (Supervisor 3, Mariga LGA)*  *The child may die (Supervisor 7, Mariga LGA)*  **Recommended supervision model by supervisors**  *Once every 2 weeks because it will be easier to prepare ourselves before going for supervision (Supervisor 1, Mariga LGA)*  *Once every 2 weeks because it would help me carry my other activities and responsibilities (Supervisor 4, Mariga LGA)*  *Once every 2 weeks because there will be more commitment, transportation money will be reduced (Supervisor 3, Mariga LGA)*  *Every week is better to ensure adequate supervision, but once every 2 weeks is okay but if there are problems the supervisors should not be blamed (Supervisor 2, Mariga LGA)*  *Once every 2weeks because of the duration of RUTF (Supervisor 5, Rijau LGA)*  *Weekly to have an accurate report (Supervisor 3, Rijau LGA)*  *Weekly because it’s a new programme (Supervisor 2, Rijau LGA)*  *Supervision should come quarterly (PM, SMOH)* |
| --- |

**Box 7. Supply chain**

| **Challenges of CORPs receiving RUTF from supervisors**  *No (challenges), because he always take RUTF stock balance during supervision visits (CORP 1, 2, 3 & 4 T/Magajiya, Rijau LGA)*  *No (challenges), because he always confirms RUTF balance during supervision visits (CORP 2, T/Magajiya, Rijau LGA)*  *No (challenges), I have enough of RUTF (CORP 3, T/Magajiya, Rijau LGA)*  *No (challenges), my supervisors is always reachable by me and he gives any commodity I request (CORP 4, T/Magajiya, Rijau LGA)*  *There was a time I experienced stock out when my supervisor travelled (CORP1, Bangi, Mariga LGA)*  *There was a time I had stock out and I experienced delay in supply (CORP1, Bangi, Mariga LGA)*  *No challenges (All CORPs, Rijau, Rijau LGA)*  *Challenges of CORPs in keeping/storing RUTF at home*  *No, we did not experience any problem we have lockable boxes (All CORPs, T/Magajiya, Rijau LGA)*  *The smell of the empty sachet attracts ant so I use insecticide powder for the ant (CORP1, Bangi, Mariga LGA)*  *Ants like the empty sachet and they come around (CORP 2, Bangi, Mariga LGA)*  *The only problem is with the ant but I’m using insecticide powder to prevent them from penetrating in to the RUTF (CORP 3, Bangi, Mariga LGA)*  *I experience the same problem of ant and I also use insecticide powder because it kills them (CORP 5, Bangi, Mariga LGA)*  *We are given a box and pad lock, all the RUTF are packed inside the box and locked it with a key, nobody has access to the key except the CORP (All CORPs, Rijau, Rijau LGA)*  **Challenges experienced by supervisors in restocking RUTF**  *There is no much challenge only that the state supervisor do not bring the RUTF on time and when it was available the CORPs supervisors were not aware (Supervisor 4, Mariga LGA)*  *Unavailability of RUTF (Stock out) (Supervisor 3, Mariga LGA)*  *Conveying the RUTF to the communities is the only challenge (Supervisor 3, Mariga LGA)*  *The funds to give the corps when they come to pick the RUTF (Supervisor 2, Rijau LGA)*  *The funds for transportation to go to the corps to restock (Supervisor 3, Rijau LGA)*  **Suggested mitigating actions by supervisors**  *Availability of commodities should be enhanced at NGO and State level (Supervisor 1, Mariga LGA)*  *Funds should be made available for transportation (All supervisors, Rijau LGA)*  *large supply of commodities during raining season to those hard-to-reach communitiesto ensure sufficient commodities. There should be proper Job description to the CHEWS(community health extension workers) to always go out to the communities to render health extension services as part of their primary duty (NGO, PM)* |
| --- |

**Box 8. Tools**

| **Difficult tools**  *Weighing scale is difficult because it gave me problem in setting it (CORP 1, T/Magajiya, Rijau LGA)*  *None of the tools was difficult to use (CORPs 2 & 4, T/Magajiya, Rijau LGA)*  *SAM registers. I always have challenge with recording number of days skipped by the defaulters whenever they come back to continue with the treatment (CORP 2, Bangi, Mariga LGA)*  *MUAC tape. When the child is not calm I find it difficult to use the MUAC tape on him/her for measurement (CORP 4, Bangi, Mariga LGA)*  *Weighing scale. I find it difficult to balance the scale to zero (CORP 5, Rijau, Rijau LGA)*  **Tools to change**  *No tool should be changed. The tools are okay, easy and simple to use No because it’s okay. (CORPs 1, 2, 3 & 4, T/Magajiya, Rijau LGA)*  *The tools are user friendly and I don’t want any change (All CORPs in Bangi, Mariga LGA)*  *No tool to change (All CORPs, Rijau, Rijau LGA)*  **Tools to add**  *The weighing scale should have a designed stand instead of hanging it on the tree (CORP 3, T/Magajiya, Rijau LGA)*  **Opinions of the supervisors/trainers/managers on the tools**  *The tools are okay, well designed, simple and easy to use (Supervisor 1, Mariga LGA)*  *The CORPs were well trained on the use of the tools, they had an initial knowledge on ICCM on the use of the Register (Supervisor 2, Mariga LGA)*  *The tools are good and simple to use but were not enough and some were not functional (Supervisor 1, Rijau LGA)*  *The corps experienced some challenges using the tools. E.g. weighing scale and MUAC tape but they are used to it (Supervisor 2, Rijau LGA)*  *There were not enough carrier bags, register and weighing scales (Supervisor 3, Rijau LGA)*  *The tools are good and very okay because they are simplified with colours and images but they should be subject to review as expected in every research (Trainer, SMOH)*  *The tools are okay, they assist the corps in their treatment, it is also a guide (PHC Director)*  *The tools are simple and common for example the weighing scale are clearly inscribed with the dosage, the dosage calculator simple and the register is well designed and simplified, they are also cheap to produce and commonly seen around (NGO, PM)*  *The tools were simple, mainly pictorialand there was no need for writing and reading, it was convinent at the community level (PM, SMOH)*  **Suggestions on improving the tools**  *The register should be made Bulkier to accommodate more names (Supervisor 1, Mariga LGA)*  *Child progress form is needed and the CORPs should be trained on how to fill it. This would enable the CORPs treat the child with or without the supervisors around (Supervisor 4, Mariga LGA)*  *The tools are ok and none should be added to avoid confusing them (Supervisor 1, Rijau LGA)*  *There should not be separate tools particularly the register for the malnutrition from that of ICCM treatment. There is need to harmonise the register to make it more easy for the corps (PM, NGO)*  *There should be IYCF guide and the counselling chart to educate the community (PM, MoH)* |
| --- |

***Box 9. Referral mechanism***

| **Reasons for non-compliance with referral advise**  *Yes some people don’t go (for referral) because the place is far and they don’t have money. We prefer to go the CORPs (Caregiver 5, Rijau, Rijau LGA)*  *Economic status of the caregivers prevents them from taking action (Supervisor 4, Mariga LGA)*  *Economic status, poverty, financial constraint and ignorance (Supervisor 1, Mariga LGA)*  *They didn’t have money to go to the referral centres (Supervisor 4, Rijau LGA)*  *Fear, they felt if the corps could not handle the case, which means the child might die (Supervisor 5, Rijau LGA)*  *They felt it was spiritual (Supervisor 3, Rijau LGA)*  *Ignorance (Supervisor 6, Rijau LGA)*  **How to improve referral compliance:**  *Words of encouragement like telling them health is wealth, every disease has treatment and they should go for the referral (Caregiver 1, Bangi, Mariga LGA)*  *Encouraging the caregivers to accept and adhere to referral (Caregiver 3, Bobi, Mariga LGA)*  *When the caregivers are assured of receiving better treatment for their children (Caregiver 7, Bobi, Mariga LGA)*  *I don’t know (Caregiver 3, T/Magajiya, Rijau LGA)*  *Let the caregiver know the importance of going to the hospital (Caregiver 2, T/Magajiya, Rijau LGA)*  *The CORP should make sure the caregiver go to the hospital by encouraging them (Caregiver 4, T/Magajiya, Rijau LGA)*  *The caregivers need to understand that if they want to save the life of their children they must adhere to the referral and go to the hospital (Caregiver 1, T/Magajiya, Rijau LGA)*  *If they are assisted with money or transport to go for the referral (Caregiver 5, Rijau, Rijau LGA*  *Creating awareness and financial mobilization to support the caregivers. There should also be community dialogue with the community members to impact on the community the benefits of these treatment (Supervisor 1, Mariga LGA)*  *Malaria consortium should give free medication note to those referral centres to encourage the caregivers since the government are refusing to take action (Supervisor 4, Mariga LGA)*  *Health talks and if the corps and supervisors have money they should assist the caregivers (Supervisor 7, Rijau LGA)*  *The corps should accompany the caregivers to the hospital and paying for them (Supervisor 6, Rijau LGA)* |
| --- |

**Box 10.** **Sustainability**

| *ICCM CORPs should be trained to treat SAM. CORPs should be motivated financially, there should be access to mobility such as provision of bicycle and motor bikes for the supervisors. The incentives provided for the CORPs should be well spread and uniform (Supervisor 1, Mariga LGA)*  ***F****inancial support should be sustained for the CORPs which would be a good motivation for them no matter the work load.* *Malaria Consortium should change referral centres from general hospital to PHC centres in the community. They should also assist and equip the PHC centres with those drugs to enable the caregivers to respond and act on referral cases and on less expenses (Supervisor 4, Mariga LGA)*  *There should be provision of commodities, equipment, and finance. On the part of the corps there is commitment already for the sustainability (Trainer, SMOH)*  *Sustainability is always a challenge, there is need for Malaria Consortium to bring council chairmen in and give them training so that they will know the importance and be committed. They should also be included in the programme. Pictures of malnourished children should be taken before and after to show progress report and it should be shown to influential leaders and council chairmen, then am sure actions would be taken up (PHC Director)*  *Malaria Consortium should continue because if the treatment is left to the Local government, there will be challenges. Hence it cant be sustained. Here should be training and retraining of corps to refresh and acquaint them more with the malnutrition treatment (PHC Director)*  *Everyone should play their own role starting from the government official i.e health workers, community supervisors and also funding to sustain the commodities and also to support the corps financially. The caregivers and other community members should be mobilised and sensitized. so in all government need to support the program, community should support the corps and the caregivers should comply (NGO, PM)*  *The state, local government should take over ownership, commit resources to the programme to ensure sustainability (NGO, PM)*  *It requires taking ownership by the government and seeing it as a responsibility and also on the part of the corps, seeing it as a responsibility to take care of their children and a collective effort by all (Clinician, Hospital)*  *For sustainability source of funding is very important. Part of the project team should arrange a report and share with the executives atleast the Governor, commissioners, showing them the results of what we have done. If the Governor decides to contribute to the project, it can be sustained. Appeal to Development partners that are willing to support projects like this (PM, SMOH)* |
| --- |
